# Crystallographic structure of the turbine *C*-ring from spinach chloroplast F-ATP synthase

**DOI:** 10.1042/BSR20130114

**Published:** 2014-03-31

**Authors:** Asha Manikkoth Balakrishna, Holger Seelert, Sven-Hendric Marx, Norbert A. Dencher, Gerhard Grüber

**Affiliations:** *Nanyang Technological University, School of Biological Sciences, 60 Nanyang Drive, Singapore 637551, Republic of Singapore; †Physikalische Biochemie, Fachbereich Chemie, Technische Universität Darmstadt, Alarich-Weiss-Str.4, D-64287 Darmstadt, Germany

**Keywords:** CF_1_F_O_-ATP synthase, *c*-rotor, crystallization, F-ATP synthase, spinach chloroplast, BN, blue native, CCP4, Collaborative Computational Project No. 4, DCCD, dicyclohexylcarbodiimid, DDM, *n*-dodecyl-β-D-maltoside, PDB, Protein Data Bank, SLS, Swiss Light Source, TM, transmembrane

## Abstract

In eukaryotic and prokaryotic cells, F-ATP synthases provide energy through the synthesis of ATP. The chloroplast F-ATP synthase (CF_1_F_O_-ATP synthase) of plants is integrated into the thylakoid membrane via its F_O_-domain subunits *a*, *b*, *b’* and *c*. Subunit *c* with a stoichiometry of 14 and subunit *a* form the gate for H^+^-pumping, enabling the coupling of electrochemical energy with ATP synthesis in the F_1_ sector.

Here we report the crystallization and structure determination of the *c*14-ring of subunit *c* of the CF_1_F_O_-ATP synthase from spinach chloroplasts. The crystals belonged to space group C2, with unit-cell parameters *a*=144.420, *b*=99.295, *c*=123.51 Å, and β=104.34° and diffracted to 4.5 Å resolution. Each *c*-ring contains 14 monomers in the asymmetric unit. The length of the *c*-ring is 60.32 Å, with an outer ring diameter 52.30 Å and an inner ring width of 40 Å.

## INTRODUCTION

The F_1_F_O_-ATP synthase is a membrane-bound multisubunit complex consisting of two rotary motors in the F_O_ and F_1_ sectors, respectively. The membrane-bound proton translocating ATP synthase of chloroplasts, CF_1_F_O_ catalyses ATP synthesis and ATP hydrolysis coupled to proton translocation across the F_O_ sector. The chloroplast F_1_ domain consists of the subunits α_3_β_3_γδε [1] and the membrane-integrated CF_O_, is made up of the subunits *a*, *b*, *b’* and *c*-ring rotor subunits, which are often named subunit IV, I, II and III, respectively [2,3]. The subcomplex α_3_β_3_ forms a hexamer with a central cavity that allows for the penetration of the γ rotor shaft [4]. Subunits γ and ε form the soluble part of the rotor shaft, called the central stalk [5]. Rotation of the central stalk subunit γ within the α_3_β_3_ cavity causes conformational changes in the three catalytic sites located at the α–β interfaces leading to ATP synthesis [6]. The two parts of ATP synthases are connected by two stalks, i.e. one central rotating shaft formed by the subunits γ and ε and a thin stalk at the periphery, composed of the subunits *b*, *b’* and δ holding together the F_1_- and F_O_ portions [5].

Subunits *a* and *c* are involved in the process of proton-pumping, thereby coupling the events of ATP synthesis and -hydrolysis in the α_3_β_3_-hexamer of F_1_. A total of ten copies of subunit *c* in yeast F-ATP synthase are found to associate into a ring structure that interacts with the foot of the central stalk subunits in the intact enzyme [7]. The copy number of *c* subunits in the ring has been experimentally studied in different organisms and is found to vary from 8 to 15 among bacterial, yeast, plant and mammalian ATP synthases [2,7–14].

The number of subunits and therefore the diameter of the proton turbine seem to be species dependent [7,8]. Atomic Force Microscopy studies, done by Seelert et al. [2] showed that the protein turbine in F_O_ of chloroplast ATP synthase has an asymmetric cylindrical structure with 14 symmetrically distributed subunits, which protrude from both membrane surfaces. Here a new protocol has been developed to obtain crystals of the *c*-ring of the spinach chloroplast F-ATP synthase, starting from the intact and active enzyme, which diffracted X-rays to 4.5 Å resolution. The crystallographic structure shows that each of the fourteen *c* subunits folds into a hairpin of two TM (transmembrane) helices, connected by a short partially structured loop. The presented *c*14-ring structure is discussed from the perspective of bioenergetic costs in plants.

## EXPERIMENTAL

### Isolation of enzymatically active chloroplast F-ATP synthase

Chloroplast ATP synthase was isolated from spinach (*Spinacea oleracea* L.) following the procedure described by Pick and Racker [15] with slight modifications as described in [16]. Thylakoid membranes were solubilized using the detergents sodium cholate (23 mM) and octyl-β-D-glucopyranoside (40 mM). For removal of contaminating proteins and lipids, the ATP synthase was subjected to fractionated ammonium sulfate precipitation and sucrose density gradient centrifugation (containing 1 mg/ml asolectin and 8 mM DDM (*n*-dodecyl-β-D-maltoside), to stabilize the protein). Thereafter, the enzyme was purified by dye-ligand chromatography with reactive red 120 [16].

### Electrophoresis

BN (blue-native)-PAGE was performed using a Hoefer Mighty Small II SE 250 system (small gel: 10 cm×8 cm×0.15 cm) as described [17,18]. The stacking gel had an acrylamide concentration of 3% and the separating gel an acrylamide gradient from 3.5 to 16%. Approximately 30 μg of solubilized purified CF_1_F_O_-ATP synthase in DDM was loaded per lane. A high molecular weight native marker (GE Healthcare) served as mass standard.

After electrophoresis the gel was scanned to document the bands stained by BN-PAGE. Subsequently, ATPase activity of the CF_1_F_O_-ATP synthase was determined by incubating the gel in buffer A1 containing 35 mM Tris, 270 mM glycine, 14 mM MgSO_4_, 0.2% (w/v) Pb(NO_3_)_2_, 8 mM ATP, pH 7.8 at 37°C for several hours [19,20]. The white lead phosphate precipitate was documented with a CCD-Imaging System (LAS 3000 intelligent dark box, Fujifilm).

SDS–PAGE was performed according to Laemmli [21], with a stacking gel of 3% and separating gel of 14%. In order to maintain integrity of the subunit *c* oligomer, protein samples were incubated at room temperature (25°C) in SDS loading buffer for 10 min. For visualization of protein bands, the gel was stained with Coomassie R-250.

### Measurement of ATP synthesis

To measure the ATP synthesis activity driven by an electrochemical proton gradient, reconstitution of solubilized ATP synthase into liposomes (phosphatidyl choline/phosphatidic acid, 9:1, w/w) was performed as previously described [20]. As a reference, CF_1_F_O_-ATP synthase was inhibited by adding DCCD (dicyclohexylcarbodiimid, 50 μM) before reconstitution into liposomes and incubation for 30 min at room temperature [22].

ATP synthesis activity of the CF_1_F_O_-ATP synthase was measured according to Fischer et al. [23] and Poetsch et al. [24] with slight modifications. 240 μl buffer L2 (200 mM Tricin, 5 mM sodium dihydrogenphosphate, 2.5 mM MgCl_2_, 120 mM KCl, 0.2 mM ADP, pH 8.3) was added into a clinicon-cuvette and mixed with 12.5 μl Luciferin–Luciferase-reagent (ATP-Monitoring Kit; Thermo Labsystems). The cuvette was placed into a Luminometer (BioOrbit 1250) and the baseline was recorded.

Approximately 43 μl of proteoliposomes were equilibrated with 217 μl buffer L1 (20 mM sodium succinate, 5 mM sodium dihydrogenphosphate, 2.5 mM MgCl_2_, 0.6 mM KCl, 1 μM Valinomycin, pH 4.7). After 100 s, the mixture was injected via a cannula into the cuvette containing L2. ATP synthesis, driven by the established TM electrochemical gradient (ΔpH and ΔK^+^), was monitored as luminescence applying the Luciferin–Luciferase-Assay. For calibrating the luminescence signal, 20 μl of 10 μM ATP was added.

### Crystallization trials of CF_1_F_O_-ATP synthase from spinach chloroplast

Initial screens of the entire CF_1_F_O_-ATP synthase at a concentration of 18 mg/ml were set up using the Memplus screen from Molecular Dimensions, UK, using the vapour diffusion method. In several drops, phase separation was observed and systematic variations of pH, precipitant and protein concentration were done to promote nucleation. Crystals grew out of the phase and took approximately 2 months to appear under optimized conditions and cryo freezed in 50% (v/v) mineral oil and paratone. Crystals were analysed at the Swiss Light Source and diffracted to 4.5 Å.

### Data collection

A single wavelength dataset of the CF_1_F_O_ crystal was collected at the protein crystallography beamline S06 PX at the SLS (Swiss Light Source) with a PILATUS 6M detector. Data were collected as a series of 0.2° oscillation images with 10 s exposure time and a detector distance of 500 mm. All diffraction data were indexed, integrated using the iMosflm program [25] and reduced with SCALA [26] and CTRUNCATE [27]. The results of data processing and data statistics are summarized in Table 1.

**Table 1:** Data collection Values in parentheses are for the highest resolution shell (6.32–6.0 Å).

Statistics of crystallographic data collection	
Wavelength (Å)	1.00
Space group	C2
Unit cell	*a*=144.420, *b*=99.295, *c*=123.51Å
	β=104.34°
Resolution range (Å)	30–6.0 (6.32-6.0)
No. of unique reflections	4238
Total no. of reflections	12041
Multiplicity	2.8 (2.8)
Completeness (%)	97.6 (98.37)
I/sigma	9.6 (5.3)
R_merge_*	0.049 (0.106)
Mosaicity	0.82

*R_merge_=ΣΣ_i_|I_h_–I_hi_|/ΣΣ_i_ I_h_, where I_h_ is the mean intensity for reflection h.

### Structure solution and refinement

The structure of the *c*-rotor of CF_1_F_O_-ATP synthase was determined by molecular replacement using the coordinates of the *c*-rotor of the proton-translocating chloroplast ATP synthase [12] [PDB (Protein Data Bank) code 2W5J]. Only the main chain atoms of the model were used for structure solution. Molecular replacement was performed using the PHASER program (TFZ=11.7, LLG score=963, [28]). Since anisotropy was observed in the data, they were subjected to ellipsoidal truncation and anisotropic scaling using the program SCALEIT, which is integrated in CCP4 (Collaborative Computational Project No. 4) [29]. Diffraction anisotropy was evidenced as a directional dependence in diffraction quality. The crystal diffracted to 4.5 Å in the horizontal direction and to 6.0 Å in the vertical direction. This kind of anisotropy is attributed to whole-body anisotropic vibration of unit cells, resulting in the crystal packing interactions being more uniform in one direction than in the other. Therefore, the R-factors were stalled at one stage during refinement and hence the data resolution was truncated at 6.0 Å. The model was manually corrected in COOT [30] and refined in REFMAC [31]. Many cycles of model building and restrained refinement with tight geometric restraints were carried using overall temperature factor and NCS (non-crystallographic symmetry) restraints. Density modification was carried out by DM in the CCP4 suite. ProSMART (Procrustes Structural Matching Alignment and Restraint Tool) was used for generation of external restraints for the refinement [32]. The PDB ID 2W5J was used as reference for exercising this restraint. In the final REFMAC cycles, TLS (translation, libration, screw) procedure was applied. The structure was validated using PROCHECK [33]. All the figures were drawn using PYMOL [34]. Structural comparison analysis are carried out using the SUPERPOSE program [35]. The atomic coordinates and structure factors have been deposited in the PDB (ID: 4MJN).

## RESULTS AND DISCUSSION

### Biochemical characterization of the entire CF_1_F_O_-ATP synthase

**Figure 1:**
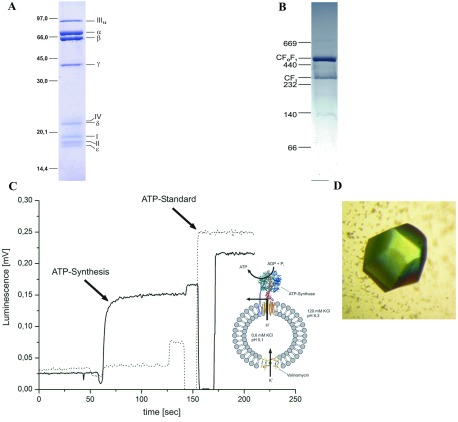
Biochemical and functional characterization of the purified chloroplast F-ATP synthase (**A**) SDS gel (14% total acrylamide and 4% cross-linked acrylamide) of the purified CF_1_F_O_-ATP synthase from spinach chloroplasts. The gel reveals the F_1_ subunits α_3_β_3_γδε and the CF_O_ subunits *b*, *b’*, *c* and *a*, respectively, which are also called I, II, III and IV in plants. Subunit *c* (III) migrates as a 14-protomer entity. (**B**) BN gel Coomassie Brilliant Blue stained of 30 μg CF_1_F_O_-ATP synthase isolated from spinach chloroplasts. (**C**) ATP synthesis activity of CF_1_F_O_-ATP synthase of *Spinacea oleracea*. The graph demonstrates the generation of ATP, induced by an electrochemical H^+^/K^+^ gradient. To demonstrate that ATP is only produced by the ATP-synthase, the measurements were also performed with CF_1_F_O_-ATP synthase inhibited by DCCD treatment before reconstitution (dotted line). (**D**) Crystal of the *c*-ring from spinach chloroplast CF_1_F_O_-ATP synthase.

The complete chloroplast F_1_F_O_-ATP synthase from spinach, used for crystallization, was characterized by different methods. The enzyme showed high purity as revealed by SDS–PAGE (Figure 1A) and BN-PAGE (1B). In the SDS gel, the bands of all subunits were present and subunit *c* (III) migrated as an intact oligomer (Figure 1B). Besides purity, the BN gel demonstrated the presence of predominantly intact CF_1_F_O_-ATP synthase (mass of>500 kDa in Figure 1B). Only a small proportion of unbound CF_1_ is present, but no CF_O_. These indicate that the crystallized *c*14-cylinder emerged from the intact enzyme. Additionally, the in-gel assay of ATP hydrolysis demonstrates the preservation of the enzymatic activity of the isolated ATP synthase preparation (results not shown). Since ATP hydrolysis in intact chloroplast ATP synthases is strictly regulated by the subunits ε and γ and other effectors [20], this activity assay can only provide a first indication of functional integrity. Therefore ATP synthesis activity of the CF_1_F_O_-ATP synthase was examined (Figure 1C). This was achieved by reconstitution of the entire CF_1_F_O_-ATP synthase into liposomes and application of an electrochemical proton gradient. With this method, an ATP synthesis activity of 41±4 ATP per enzyme per second was determined.

### Crystallization and preliminary X-ray analysis

The enzymatically active and entire CF_1_F_O_-ATP synthase was used for crystallization and crystals of good diffraction quality appeared from the optimized phase separated drop under conditions of 33% (v/v) PEG 550 MME, 0.05 M sodium acetate pH 4.3 (Figure 1D). The crystals were colourless, in contrast to recently obtained yellow crystals, which were described to contain one chlorophyll and two carotenoids [3]. However, no protein structure from this crystal form has yet been described [3].

The crystals obtained diffracted to 4.5 Å resolution and belonged to C2 space group (β=104.34°), with unit-cell parameters *a*=144.42, *b*=99.29, and *c*=123.51 Å. The phases were obtained using the crystal structure of the *c*14 rotor ring of the chloroplast F-ATP synthase (PDB ID 2W5J [12]. The solvent content was calculated to be 63.45% and *V_m_* was 3.36 Å^3^ per Da [36].

Analysis of the data as well as the solution from molecular replacement confirmed that only the *c*-ring rotor of the chloroplast ATP synthase is present in the crystal structure, whereas all the F_1_ subunits α, β, γ, δ and ε and subunits *a*, *b* and *b’* of the membrane-integral F_O_ domain are lost during the crystallization process. The initial electron density map also suggests that most probably only the *c*-ring is present in the crystal structure.

### Overall structure of the *c*-rotor

**Figure 2:**
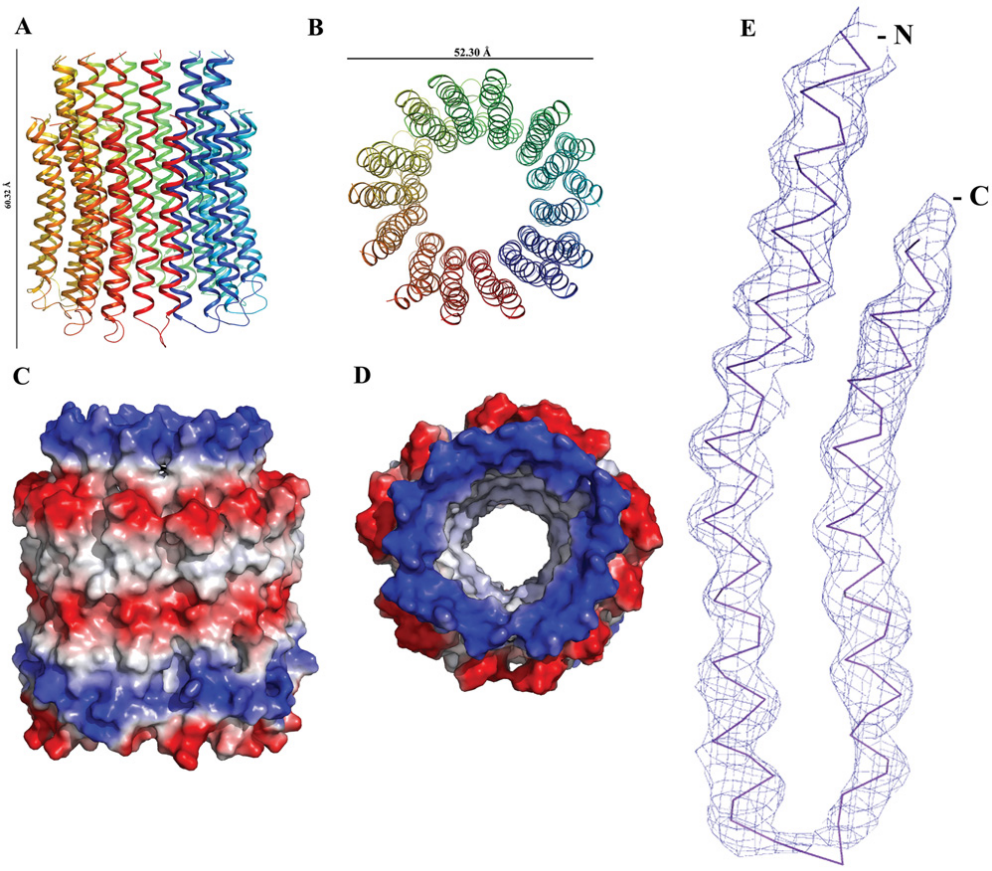
The structure of the *c*-ring of the spinach chloroplast F-ATP synthase (**A**) Cartoon representation of the *c*-ring in side view and (**B**) view from the lumen. (**C**) The electrostatic potential (calculated in pymol [34]) mapped on the surface of the *c*-ring with positive areas in blue, negative in red, and neutral in white colour, in side view. (**D**) The electrostatic potential mapped on the surface of the *c*-ring, the view is from the lumen. (**E**) Electron density map (2F_o_-F_c_) of chain A at 1σ for the *c*-ring.

**Figure 3:**
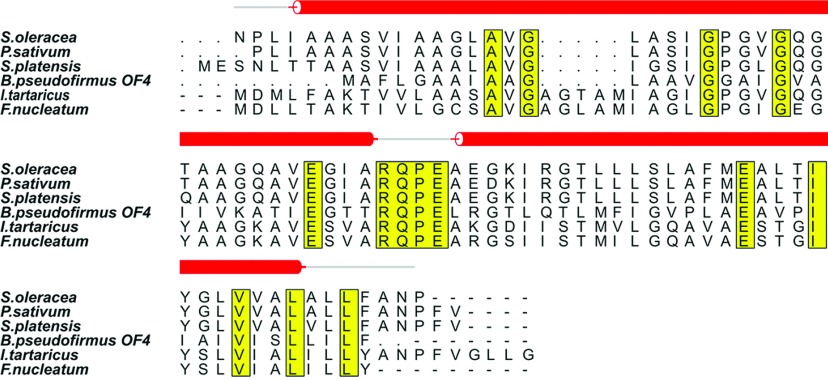
Multiple sequence alignment of *c subunits* of F-ATP synthases from *Spinacia oleracea, Pisum sativum, Spirulina platensis, Bacillus pseudofirmus OF4, Ilyobacter tartaricus and Fusobacterium nucleatum* All the conserved residues are highlighted in yellow. The sequence of Spinach chloroplast *c* subunit along with its secondary structure was aligned to related homologous structures. The secondary structures are drawn in red. The figure was generated using ALSCRIPT.

The structure of the *c*-rotor of the spinach chloroplast F-ATP synthase was determined by the molecular replacement method and refined to 6.0 Å. A summary of the refinement statistics is given in Table 2. Analysis of the stereochemical quality of the final model by PROCHECK has identified that 97% of all the residues are within the core regions of the Ramachandran plot, 2.1% are within the allowed regions, and 0.9% are within the generously allowed region. No residues are present in the disallowed region. The last four C-terminal amino acids are not visible in the electron density map. Validation with ERRAT server gave an overall quality factor of 97.03% [37]. The rmsd (root-mean-square distance) between the presented structure and the previously determined *c*-ring of the chloroplast ATP synthase (2W5J [12]) is 1.509 Å. The overall structure of the spinach chloroplast *c-*ring is shown in Figures 2(A) and 2(B). Each subunit *c* folds into a hairpin of two TM helices, connected by a short partially structured loop (Figure 2E). The individual hairpin, composed of an N-terminal TM and a C-terminal TM, produce an inner- and an outer ring. The N-terminal helices face the inner side of the ring, whereas the C-terminal helices face the outside of the loop. This arrangement facilitates interaction of the conserved glutamic acid residues of subunit *c* (Figure 3) with the conserved Arginine residue from subunit *a*, providing the structural requirements to form a part of the proton channel in the F_O_ domain. Figures 2(C) and 2(D) display the electrostatic charge surface of this rotary entity. The cytoplasmic and the luminal surfaces of the *c* ring are very polar in nature as they are in contact with the aqueous phase. The hydrophilic patch at the centre of the membrane region represents the proton-binding site.

**Table 2:** Refinement statistics

Refinement statistics	
*R factor(%)	35.3
^†^R free(%)	37.8
Ramachandran statistics	
Most favored (%)	97
Additionally allowed (%)	2.1
Generously (%)	0.9
Disallowed (%)	0.0
R.M.S. deviations	
Bond lengths (Å)	0.019
Bond angles (°)	2.019
Mean atomic B values (Å^2^)	
Overall	39.72

*R-factor=Σ∥F_O_|–|F_C_∥/Σ|F_O_|, where F_O_ and F_C_ are measured and calculated structure factors, respectively.

^†^R-free=Σ∥F_O_|–|F_C_∥/Σ|F_O_|, calculated from 5% of the reflections selected randomly and omitted from the refinement process.

The length of the spinach chloroplast *c-*ring assembly is 60.32 Å long. The outer ring diameter is 52.3 Å, and the inner ring has a width of 40 Å. The inner space would be wide enough to accommodate detergent molecules. Interestingly, electron density could be observed in the initial maps in this region. However, this cannot be confirmed at this moderate resolution. Previously studies of 2D crystals of the intact CF_O_ or *c*_14_*a* complex by AFM and Cryo-electron microscopy revealed distinct mass/electron density inside and protruding from the *c*14-cylinder. The isolated *c*14-cylinder, however, was ‘empty’ [41,42]. The electron density for the hairpin was continuous. Because of the achieved resolution, only the density for the main chains could be identified (Figure 2E).

The crystal lattice reveals one *c*-ring consisting of 14 monomers (*c*14 ring) in the asymmetric unit forming crystal contacts with three neighbouring rings. The κ=180° section of the self-rotation function, calculated for the crystal form using MOLREP (Collaborative Computational Project No. 4) with an integration radius of 20.0 Å and data in the resolution range 15–6 Å, showed a string of 14 peaks. The loop region of one ring is in close contact with the loop region of its symmetry mate. The other two points of contact in the crystal lattice involves the monomers in the N-terminal region.

### Comparison with *c*-rotors of other organisms

Crystallographic structures of the *c*-ring rotors have been reported for F-ATP synthases from *Ilyobacter tartaricus* [9], *Spirulina platensis* [13], *Pisum sativum* [38] and *Saccharomyces cerevesiae* [7,10]. Among all the *c*-ring structures, only the spinach and pea chloroplasts are related by tetradecameric symmetry in the asymmetric unit. Despite this similarity, the two display markedly different shapes, with the *c*-ring of *P. sativum* taking up a concave barrel shape with a pronounced waist in the middle, whereas that of spinach chloroplast has a much less pronounced concave curvature.

The diameters of these *c*-ring rotors differ from organism to organism, because of the variation in the number of protomers. The undecameric *c*-ring of *I. tartaricus* shows a cylindrical, hourglass shaped protein complex with an outer diameter of ~50 Å and inner diameter of ~17 Å [9]. The pea chloroplast *c*-ring has an outer ring diameter of 60.5 Å and an inner ring diameter of 35 Å [38]. The *c*15 ring from *S. platensi*s has an hour glass shaped assembly with an outer diameter of 65 Å and an inner diameter of 54 Å [13]. The yeast *c*-ring diameter in the F_1_*c*10 complex structure is ~55 Å and the inner diameter is ~27 Å and comprises ten protomers [7]. In comparison, the spinach chloroplast *c*14 ring presented here has an external diameter of 52.30 Å, whereas that of the inner ring is 40 Å. From the perspective of bioenergetic costs 14 protons have to be translocated across the thylakoid membrane per full rotation of the rotary *c*-ring, whereby each rotation of 360° produces three molecules of ATP in the α_3_β_3_-headpiece of F_1_. Therefore 3.7 protons are needed to synthesize one molecule of ATP in the spinach chloroplast F-ATP synthase. In comparison, 2.7 protons per ATP are required for the bovine mitochondrial F-ATP synthase, whose *c*-ring consists of eight *c* subunits [8], reflecting a more efficient enzyme with respect to protons consumed per ATP generated, at least at first glance. However, the two components of the proton-motive force, i.e. the membrane potential and the pH gradient, are kinetically not equivalent and different sizes of the cation-powered rotors may be nature's solution to cope with the challenges of diverse environments [42].

Recently, two more *c*-ring crystal structures have been solved, one from *Fusobacterium nucleatum*, which has 11 protomers [39], and the other from *Bacillus pseudofirmus OF4*, which has 13 protomers [40]. These studies demonstrated that the alanine motif in the *c*13-ring from *B. pseudofirmus OF4* contributes to high complex stability, with A-to-G mutations reducing the *c*-ring stoichiometry, in this case from *c*13 to *c*12, whereas G-to-A mutations increases it. From the sequence alignment in Figure 3, it can be seen that the *c*11-ring of *I. tartaricus* consists of 17.8% alanine residues compared with an alanine content of 21.8% in the *c*14-ring of the spinach chloroplast F-ATP synthase investigated here.

There is urgent need to determine the structure of the intact CF_O_ turbine, in order to elucidate the spatial arrangement of the subunits *b* and *b’*, and especially of subunit *a* in relation to the *c*14-ring. This would also lead to an answer to the pertinent question of how the *c*-ring is made proton-tight so as to prevent harmful proton leaks across its central structure, that would otherwise bypass the specific proton pathway between subunit *a* and the *c* subunits.
